# Anticipatory Baseline Pupil Diameter Is Sensitive to Differences in Hearing Thresholds

**DOI:** 10.3389/fpsyg.2019.02947

**Published:** 2020-01-10

**Authors:** Nicolai D. Ayasse, Arthur Wingfield

**Affiliations:** Volen National Center for Complex Systems, Brandeis University, Waltham, MA, United States

**Keywords:** hearing acuity, pupil dilation, baseline pupil dilation, aging, listening effort

## Abstract

Task-evoked changes in pupil dilation have long been used as a physiological index of cognitive effort. Unlike this response, that is measured during or after an experimental trial, the baseline pupil dilation (BPD) is a measure taken prior to an experimental trial. As such, it is considered to reflect an individual’s arousal level in anticipation of an experimental trial. We report data for 68 participants, ages 18 to 89, whose hearing acuity ranged from normal hearing to a moderate hearing loss, tested over a series 160 trials on an auditory sentence comprehension task. Results showed that BPDs progressively declined over the course of the experimental trials, with participants with poorer pure tone detection thresholds showing a steeper rate of decline than those with better thresholds. Data showed this slope difference to be due to participants with poorer hearing having larger BPDs than those with better hearing at the start of the experiment, but with their BPDs approaching that of the better hearing participants by the end of the 160 trials. A finding of increasing response accuracy over trials was seen as inconsistent with a fatigue or reduced task engagement account of the diminishing BPDs. Rather, the present results imply BPD as reflecting a heightened arousal level in poorer-hearing participants in anticipation of a task that demands accurate speech perception, a concern that dissipates over trials with task success. These data taken with others suggest that the baseline pupillary response may not reflect a single construct.

## Introduction

Although the primary biological function of flexibility in the size of the pupil of the eye is to modulate the amount of light reaching the retina (Wang et al., 2016), pupil size also responds to a range of psychological states (e.g., Kim et al., 2000; Kinner et al., 2017). Since the 1960s the measurement of task-related changes in pupil dilation (*pupillometry*) has been used as an objective index of cognitive effort. This use followed from the demonstration that a progressive increase in the difficulty or effort required to complete a cognitive task is accompanied by a progressive increase in pupil dilation (Hess and Polt, 1964; Kahneman and Beatty, 1966; see reviews in Beatty and Lucero-Wagoner, 2000; van der Wel and van Steenbergen, 2018).

In the domain of speech comprehension, the relationship between cognitive effort and pupil dilation appears in the form of an increase in pupil diameter while listeners are attending to speech degraded by noise or reduced hearing acuity (e.g., Zekveld et al., 2011; Koelewijn et al., 2012; Kuchinsky et al., 2014; Ayasse et al., 2017; Ayasse and Wingfield, 2018), or when listeners are faced with sentences that express their meaning with complex syntax (Piquado et al., 2010; see Zekveld et al., 2018, for a review). In studies such as these, task-related changes in pupil diameter are typically expressed as a difference from a pre-stimulus baseline in the form of a brief silent period prior to presentation of the speech stimulus. The pupil size measured in this pre-stimulus window is referred to as the baseline pupil diameter (BPD; Kuchinsky et al., 2013; Koelewijn et al., 2015; Winn and Moore, 2018; Wagner et al., 2019).

Although studies in cognitive pupillometry traditionally focus on the change in pupil size relative to baseline (the *task-evoked pupillary response*; see Reilly et al., 2018), several studies have focused on the BPD itself. Unlike the task-evoked pupillary response that is measured during or after an experimental trial, the BPD is a measure taken prior to an experimental trial. As such, the BPD can be seen as a measure of an individual’s anticipatory mental state at that particular moment in an experiment.

Since the introduction of the Yerkes–Dodson Law (Yerkes and Dodson, 1908) there has been a recognition of the relationship between level of arousal and level of performance. That is, the quality of performance will be poor in the case of fatigue or reduced task engagement (low arousal), and poor at the other extreme, when there is anxiety or stress (high arousal), with optimal performance expected at a level of arousal between these two extremes (Stennet, 1957; Kahneman, 1973; see also the discussion in McGinley et al. (2015) and Pichora-Fuller et al., 2016). Following this principle, when BPD has been considered in cognitive tasks, the possibility has been raised that a cognitively demanding experimental task that causes a build up of mental fatigue may result in a decrease in physiological arousal, reflected in turn by a decrease in BPD. The result of this postulated chain would be a decrease in task engagement, with a resultant decline in task performance over the course of the experiment (e.g., Murphy et al., 2011; Hopstaken et al., 2015; Winn et al., 2018).

A potential decrease in BPD over the course of an experiment takes on special significance in the context of speech recognition in individuals with hearing impairment, who often report mental fatigue consequent to the need for effortful listening (Hasson et al., 2009; Nachtegaal et al., 2009; Hornsby et al., 2016). To the extent that a decrease in BPD over the course of an experiment is indicative of participant fatigue, one might predict that individuals with hearing impairment would show a stronger fatigue effect than those with better hearing. That is, one would expect to see for individuals with poor hearing a steeper slope in a plot of BPD over the course of an experiment compared to those with better hearing.

Although as noted several studies using speech stimuli have examined effects of hearing impairment on task-related pupil dilation, few studies have examined effects of hearing on the BPD itself. Those that have, have produced mixed results (cf., Zekveld et al., 2011; Kramer et al., 2016; Wang et al., 2018).

In order to address the question of whether those with poorer hearing would show a steeper decrease in BPD across a testing session, we drew BPD data from a larger ongoing study of speech comprehension. We aimed to determine (a) whether one will observe a progressive decrease in BPD over the course of a large number of listening trials, and, (b) to the extent this is so, whether participants with hearing impairment may exhibit a differentially steeper decrease in BPD over the course of the listening trials than those with better hearing as measured by pure tone detection thresholds. Our final question is whether decreasing performance accuracy is an invariant concomitant of decreasing BPD, and with it the presumption of a unified account of the meaning of BPD in behavioral experiments.

## Materials and Methods

### Participants

Participants were 68 adults (46 female, 21 male, one chose not to disclose) ranging in age from 18 to 89 years (*M* = 60.0, *SD* = 26.8). Participants were drawn from university students, staff, and volunteers from the local community.

Audiometric evaluation was carried out for each participant using an AudioStar Pro clinical audiometer (Grason-Stadler, Inc., Madison, WI, United States) by way of standard audiometric techniques in a sound-attenuated testing room. The participants’ better-ear pure tone average (PTA) over 0.5, 1, 2, and 4 kHz ranged from 1.3 to 50.0 dB HL (*M* = 21.0, *SD* = 12.8). Participants’ speech reception thresholds (SRT) in quiet (measured via recorded 36-word CID W-1 spondees; Rourke-Cullen et al., 1995) ranged from 5.0 to 62.5 dB HL (*M* = 22.7, *SD* = 11.7). None of the participants were regular users of hearing aids and all testing was conducted unaided. All participants reported themselves to be native speakers of American English, with no history of stroke, Parkinson’s disease, or other neurologic involvement that might compromise their ability to perform the research task. Written informed consent was obtained from all participants according to a protocol approved by the Brandeis University Institutional Review Board.

### Stimuli and Procedures

Stimuli consisted of 12- to 14-word sentences that contained an agent, an action, and the recipient of the action. The sentences were recorded by a female speaker of American English onto computer sound files using Sound Studio v2.2.4 (Macromedia, Inc., San Francisco, CA, United States) that digitized (16-bit) at a sampling rate of 44.1 kHz. Root-mean-square (RMS) amplitude was equated across stimuli.

Participants heard a total of 160 sentences presented binaurally via Eartone 3A insert earphones (E-A-R Auditory Systems, Aero Company, Indianapolis, IN, United States) at 20 dB above each participant’s better-ear SRT (i.e., 20 dB Sensation level; *SL*). After each sentence was presented, the participant was asked to indicate whether a given character was the agent of the action or the recipient of the action. A trial consisted of a 60 s baseline silent period followed by the presentation of a recorded sentence and then the participant’s response.

To ensure audibility of the stimuli, participants were asked to repeat single words recorded by the same speaker and presented at the sound level to be used in the main experiment. All participants passed this audibility screen with 100% accuracy.

### Pupillometry

Throughout the course of each trial the participant’s moment-to-moment pupil size was recorded via a desk-mounted EyeLink 1000 Plus eye-tracking apparatus (SR Research, Oakville, ON, Canada), using a standard 9-point calibration procedure. The Eye-Link acquired pupil size data at a rate of 1000 Hz, with data recorded via MATLAB software (MathWorks, Natick, MA, United States).

Blinks were removed, and linear interpolation was performed starting 80 ms before and ending 160 ms after each blink (e.g., Zekveld et al., 2010, 2014; Wendt et al., 2016). This procedure was employed to reduce artifacts resulting from partial closures of the eyelids at the beginning and ending of a blink that would cause partial obscurations of the pupil (Siegle et al., 2008; Winn et al., 2015, 2018). To aid in accurate pupil size measurement, the participant’s head was stabilized using a customized chin rest, and participants were asked to keep their eyes on a centrally located fixation point continuously displayed on a computer screen placed over the EyeLink camera. The ambient lighting in the testing room was maintained at a constant level throughout the experiment.

#### Adjustment for Senile Miosis

Older adults generally exhibit an overall smaller pupil size and a smaller range of excursion when compared to younger adults (*senile miosis;* Bitsios et al., 1996). This generality appeared in our present sample. The mean raw baseline pupil diameter for participants below 60 years old was 49.0 AUs (*SD* = 19.8; values given in arbitrary units, AUs, standard to EyeLink trackers; e.g., Papesh and Pinto, 2019), and the mean raw BPD for participants at or above 60 years old was 29.8 AUs (*SD* = 6.4). *Pupillary dynamic range* was estimated by measuring each participant’s change in pupil dilation in response to light (199.8 cd/m^2^) and dark (0.4 cd/m^2^) screens presented for 60 s each. The mean dynamic range for participants below 60 years old was 26.1 AUs (*SD* = 7.4) and for participants above 60 years old was 14.9 AUs (*SD* = 6.3).

As would be expected (Bitsios et al., 1996), participants’ raw BPD was significantly correlated with their age, *r*(66) = −0.60, *p* < 0.001, with older adults showing a significantly smaller raw BPD than the younger adults. There was also a correlation between raw BPD and individuals’ PTA, *r*(66) = −0.56, *p* < 0.001. However, a partial correlation conducted between raw BPD and PTA, partialing out the effects of age, was not significant, *r*(66) = −0.13, *p* = 0.299, while the partial correlation between raw BPD and age, partialing out the effects of PTA, remained significant, *r*(66) = −0.29, *p* < 0.017. Also as would be expected (Bitsios et al., 1996), participants’ pupillary dynamic range was significantly correlated with their age, *r*(66) = −0.72, *p* < 0.001, with older adults showing a smaller dynamic range than younger adults. There was also a correlation between pupillary dynamic range and individuals’ PTA, *r*(66) = −0.62, *p* < 0.001. However, a partial correlation conducted between pupillary dynamic range and PTA, partialing out the effects of age, was not significant, *r*(66) = −0.03, *p* = 0.791, while the partial correlation between pupillary dynamic range and age, partialing out the effects of PTA, remained significant, *r*(66) = −0.48, *p* < 0.001. That is, for this sample, the differences in raw BPD and pupillary dynamic range were driven by age, not by hearing.

To adjust for this age difference, baseline pupil sizes were scaled by representing pupil dilation as a proportion of each participant’s dynamic range (e.g., Allard et al., 2010; Piquado et al., 2010; Ayasse et al., 2017; Ayasse and Wingfield, 2018). It should be noted that this method assumes that age-related changes to the pupil’s response to light are proportional to age-related changes to the pupil’s response to arousal level or cognition; however, no method that avoids such an assumption has yet been standardized (see also discussions in Winn et al., 2018; Zekveld et al., 2018).

The BPD was calculated as the mean pupil size measured over the last 30 s of the 60 s silent period prior to each sentence, represented as a proportion of the individual’s pupillary dynamic range.

## Results

### Change in Baseline Pupil Dilation Over Trials

**FIGURE 1:**
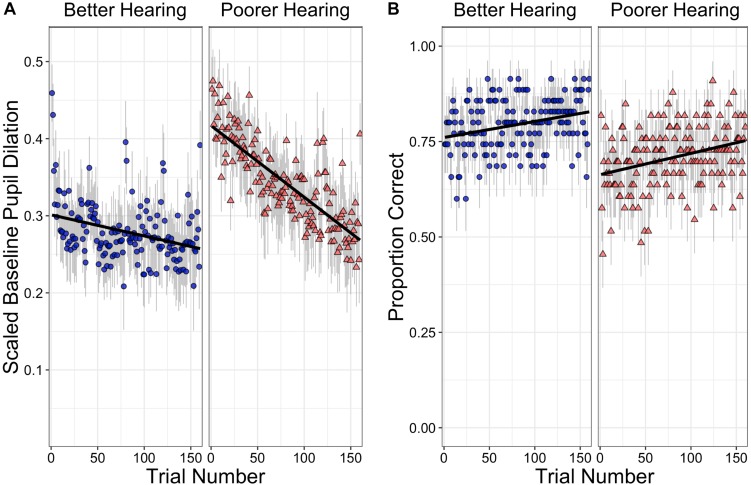
**(A)** shows the change in baseline pupil dilation (BPD) over the course of the listening task for participants with better hearing **(left)** or poorer hearing **(right)**. For this illustration, hearing groups were based on a median split of pure tone thresholds (PTAs), with PTAs for the better hearing group ranging from 1.3 to 21.3 dB HL (*M* = 10.9), and the poorer hearing group ranging from 22.5 to 50.0 dB HL (*M* = 31.4). BPD is shown as a proportion of participants’ dynamic range. **(B)** shows the proportion of correct comprehension responses over the course of the listening task for the two ranges of hearing thresholds. Error bars in both figures represent the standard error of the mean for a given trial.

Although hearing sensitivity as measured by individuals’ PTA is treated as a continuous variable in the analysis that follows, the effect of PTA on BPD is illustrated in Figure 1A that shows scaled BPDs over the course of the 160 trials for participants with better and with poorer hearing. For this illustration, hearing groups were based on a median split of PTAs, with PTAs for the better hearing group ranging from 1.3 to 21.3 dB HL (*M* = 10.9), and the poorer hearing group ranging from 22.5 to 50.0 dB HL (*M* = 31.4). The better hearing group thus fell within the range considered clinically normal hearing for speech (PTA < 25 dB HL; Katz, 2002), while the participants in the poorer hearing group ranged from slight-to-moderate hearing loss.

It can be seen that although both groups show a linear decline in BPD over the course of the experimental trials, the participants with poorer hearing acuity show a much steeper decline over the course of the experiment than those with better hearing acuity. It can also be seen, however, that this steeper decline is consequent to the poorer hearing participants showing a larger BPD at the start of the experiment than the better hearing group, but ending at a similar level as those with better hearing.

As would be expected from population demographics, the present sample reflected a general relationship between age and individuals’ PTA, *r*(66) = 0.83, *p* < 0.001. For this reason, and to represent PTA and age as continuous variables, a linear mixed-effects model was run with BPD serving as the dependent variable. Participants and sentence items were included as random effects, such that by-participant and by-item random intercepts and random slopes were included for the continuous variables of PTA, age, and trial number (Barr et al., 2013). All variables were scaled and centered before being added into the model using the *scale* function. The analysis was carried out in R version 3.5.2 using the *lme4* package (version 1.1-19) and the function *lmer* to fit the model. The effects of each variable on model fit were evaluated using model comparisons of the change in log-likelihood. Fixed effects and interactions are shown in the Predictor column and were added in the order they are listed in the table. Coefficients and *X*^2^ test results are shown for each step of the model.

As seen in Table 1, there were significant main effects of trial number and participant PTA on BPD, as well as a significant Trial number × PTA interaction indicative of the slope differences over trials as a function of PTA. There was also a significant main effect of age, but age did not have a significant effect on the change in BPD over time, reflected in the absence of a significant Trial number × Age interaction. The remaining significant effect in the model was a significant three-way Trial number × Age X PTA interaction, indicating that age and PTA had a multiplicative effect on the rate of BPD decline across trials. (When age was entered into the model first, the major patterns shown in Table 1 remained the same.)

**TABLE 1:** Linear mixed-effects model of continuous variables for baseline pupil dilation (BPD).

**Predictor**	***B*^a^**	**χ^2^^b^**	***df*^c^**	***p*^d^**
Trial number	–0.11	200.86	1	**<0.001^∗∗∗^**
PTA	0.24	6.34	1	**0.012^∗^**
Trial number × PTA	–0.04	54.28	1	**<0.001^∗∗∗^**
Age	0.34	4.08	1	**0.043^∗^**
Trial number × Age	–0.02	3.02	1	0.082
PTA × Age	0.21	1.29	1	0.256
Trial number × PTA × Age	0.03	9.09	1	**0.003^∗∗^**

*Significant *p* values are bolded. ^*a*^Unstandardized coefficient (of standardized variables). ^*b*^χ^2^ value for comparisons of each step of the model. ^*c*^Degrees of freedom for the χ^2^ test. ^*d*^*p* value reflects significance of change in model fit at each step of the model. ^∗^*p* < 0.05, ^∗∗^*p* < 0.01, ^∗∗∗^*p* < 0.001.*

### Response Accuracy

If a decline in BPD over trials is to be considered as a reflection of a decrease in arousal concomitant to an increase in fatigue and a decline in task engagement, one is bound to ask whether there is a relationship between BPD and participants’ comprehension accuracy. Specifically, one might ask whether performance accuracy declines over the course of the task along with the observed decline in BPD. Figure 1B shows a plot of accuracy over trials using the same median split according to hearing acuity as used in Figure 1A. It can be seen that, although BPD declined across trials, accuracy in fact shows a slight improvement over the course of the 160 trials for participants with both good and poor hearing.

## Discussion

There is a general consensus in the literature that the BPD reflects an individual’s current state of arousal (Antikainen and Niemi, 1983; Aston-Jones and Cohen, 2005; Zekveld et al., 2010, 2018; Hopstaken et al., 2015), with one’s level of arousal increased with positive motivation (Kahneman, 1973; Pichora-Fuller et al., 2016), and decreased by cognitive fatigue (Hopstaken et al., 2015). Consistent with this view, Hopstaken et al. (2015) found a decline in BPD accompanied by a decline in performance over the course of 189 trials of an N-back task (hearing a continuous stream of random letters and at each point quickly naming the letter that had occurred 1, 2 or 3 items previously). Interpreting these results in terms of fatigue developing over trials would seem a reasonable conclusion given the multiple cognitive demands of the N-back task and its novelty to everyday experience (Kane et al., 2007).

It might be tempting to similarly interpret the progressive decline in BPD over the course of the 160 comprehension trials in the present study as reflecting participant fatigue. It would further follow from this argument that, because participants with hearing impairment must engage more resource-demanding effort for front-end perception, a buildup of fatigue and consequent reduction in task engagement would lead to the observed steeper decline in BPD over the course of experimental trials relative to those with better hearing.

Inconsistent with a fatigue argument, however, was the finding that, although a steeper decline in BPD appeared over trials for those with poorer hearing, accuracy in the sentence comprehension task increased over trials rather than decreased. It was also seen that the steeper rate of decline in BPD for those with poorer hearing relative to those with better hearing was in fact due to the poorer-hearing participants having larger BPDs at the beginning of the experiment, and then beginning to approximate those of the better-hearing participants after experience with the task.

It has been over a 100 years since the articulation of the Yerkes–Dodson Law (Yerkes and Dodson, 1908; see also McGinley et al., 2015), yet it still may account for several apparent conflicts in the literature as well as for our own data. As indicated, some papers that have examined BPD have related a smaller baseline pupil size, and presumed lower arousal, to fatigue when this is accompanied by poorer performance (e.g., Hopstaken et al., 2015). Other papers have related a larger baseline pupil size, and presumed higher arousal, to an over-aroused state when this is accompanied by poorer performance (e.g., Gilzenrat et al., 2010). That is, it can be argued that the former case is looking at the left half of the classic Yerkes–Dodson curve, going from low arousal and poor performance to optimal arousal and improved performance. The latter case, and our data, is looking at the right half of the classic Yerkes–Dodson curve, going from over-arousal and poor performance to optimal arousal and improved performance.

We assume, along with the extant literature, that BPD reflects the individuals’ level of arousal at that point at which it is being measured, in this case the time window prior to a sentence being presented. We suggest that, to the extent that BPD indexes the individual’s anticipatory mental state, individuals with poorer hearing would enter a task that relies on accurate speech perception with an increased level of anticipatory arousal than those with better hearing. One would thus expect to see this early level of anticipatory arousal to decrease progressively due to increasing success as the task of determining agents and recipients of actions in sentences improved with practice.

It should be recognized that the poorer-hearing participants’ larger BPDs in the early experimental trials is open to at least two alternative interpretations, both of which were forecast by Kahneman (1973; see also Pichora-Fuller et al., 2016), and both of which are consistent with the present data. The first is that the higher level of arousal in the early experimental trials by the individuals with poorer hearing could reflect a heightened increase in attentional allocation as these participants readied themselves to deal with an anticipated need for effective perception of spoken materials. The second possibility is that participants with poorer hearing might show an increased level of arousal reflecting task anxiety or a lack of confidence in likely success (e.g., Lempert et al., 2015). Either of these two states would be seen to have dissipated as the experiment proceeded and the task was, and was perceived to be, less challenging than at the start. The observed interaction between hearing and age on the slope of the BPD decrease suggests that older age slightly compounds this effect, maybe due to additional factors such as cognitive or higher-level auditory processing changes that are subsumed under age differences in the current study.

We do not intend to deny that a decline in pupil size over the course of an experiment can in some cases reflect a decline in task engagement linked to fatigue. As we have noted, such a finding has been reported for the especially taxing N-back task (Hopstaken et al., 2015). Rather, we suggest that it might be a false economy to seek a single account of BPD that necessarily applies across all tasks.

## Data Availability Statement

The datasets generated for this study are available to any qualified researcher upon request to the authors.

## Ethics Statement

The studies involving human participants were reviewed and approved by Brandeis University Institutional Review Board (IRB). The participants provided their written informed consent to participate in this study.

## Author Contributions

NA and AW collaborated on the experimental design, data analysis, data interpretation, and drafting of this manuscript.

## Conflict of Interest

The authors declare that the research was conducted in the absence of any commercial or financial relationships that could be construed as a potential conflict of interest.
